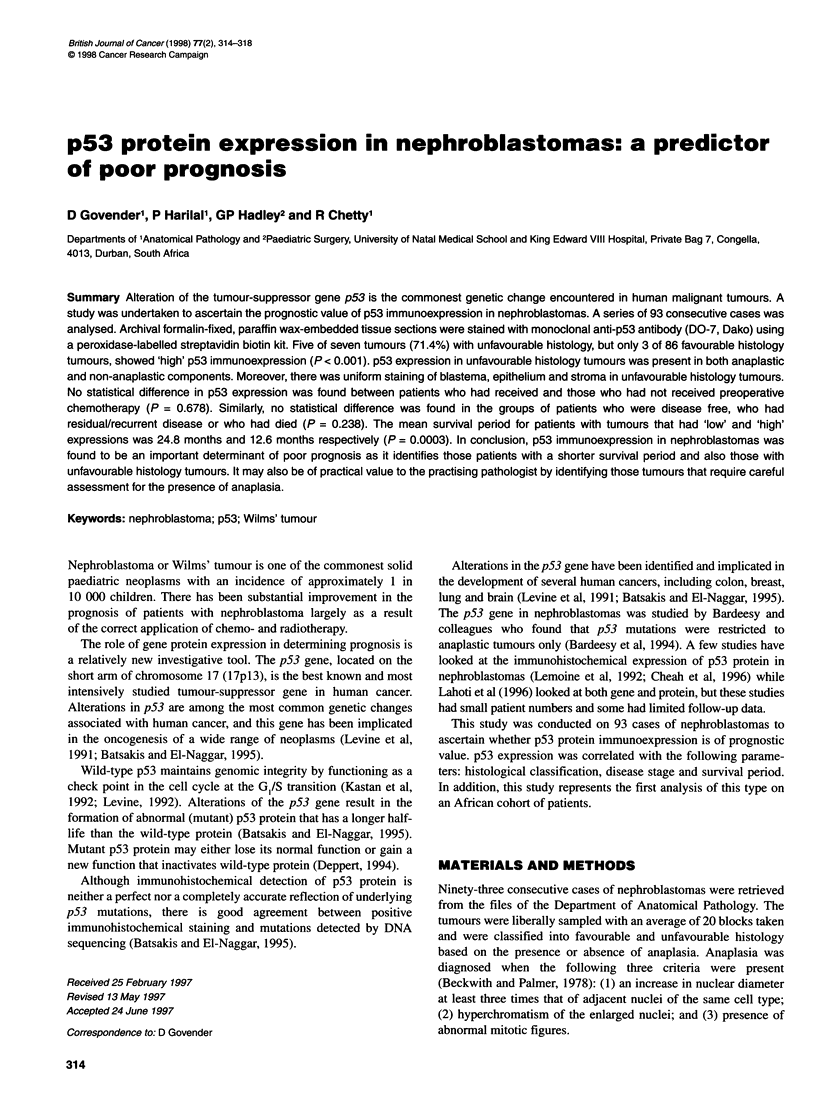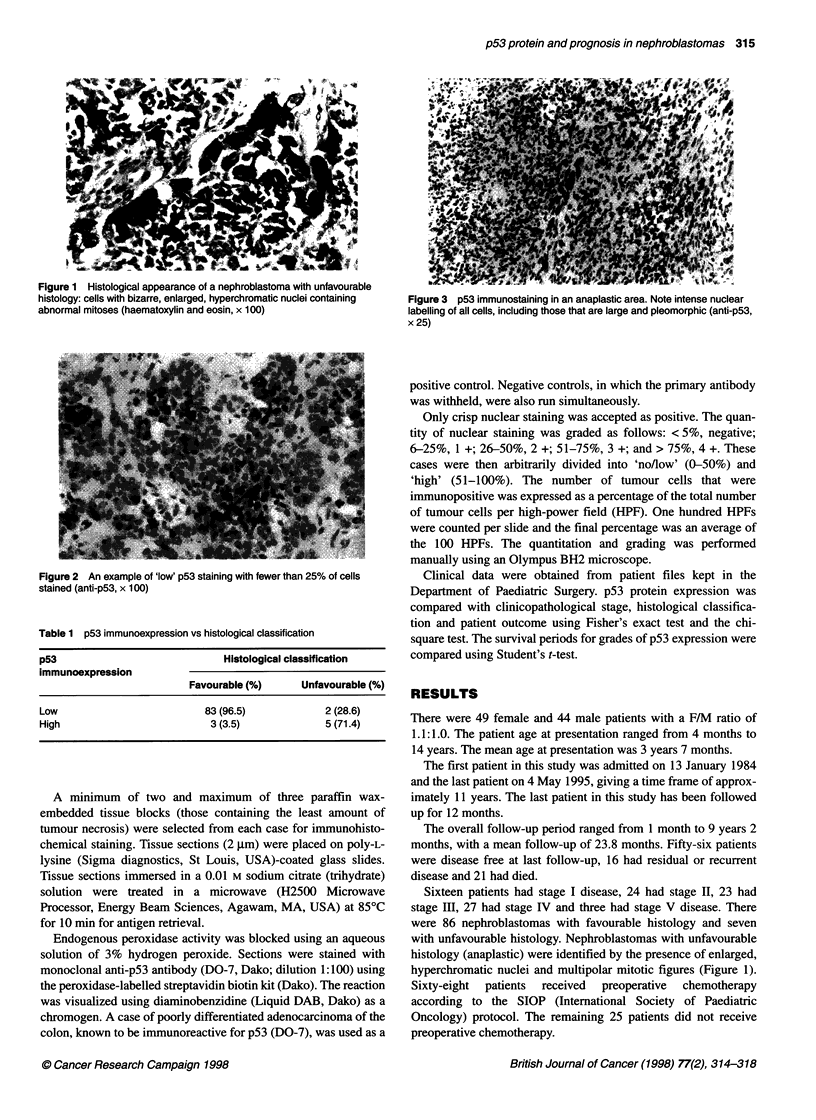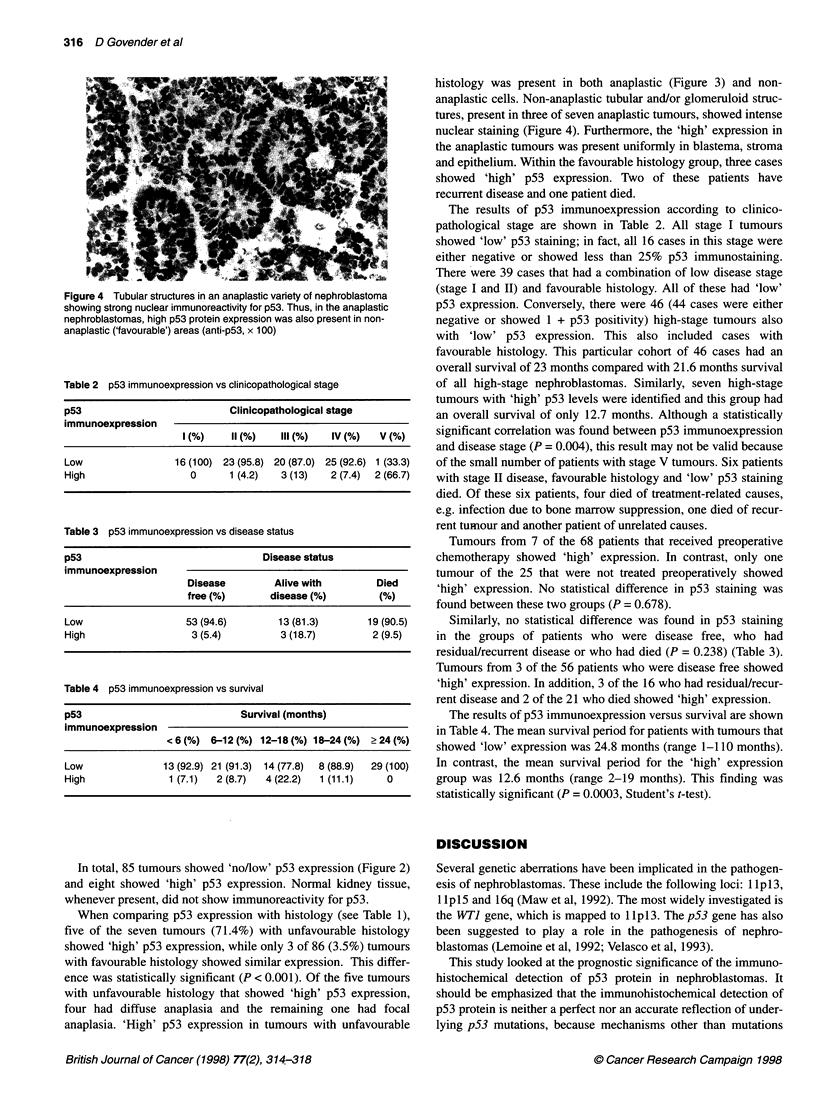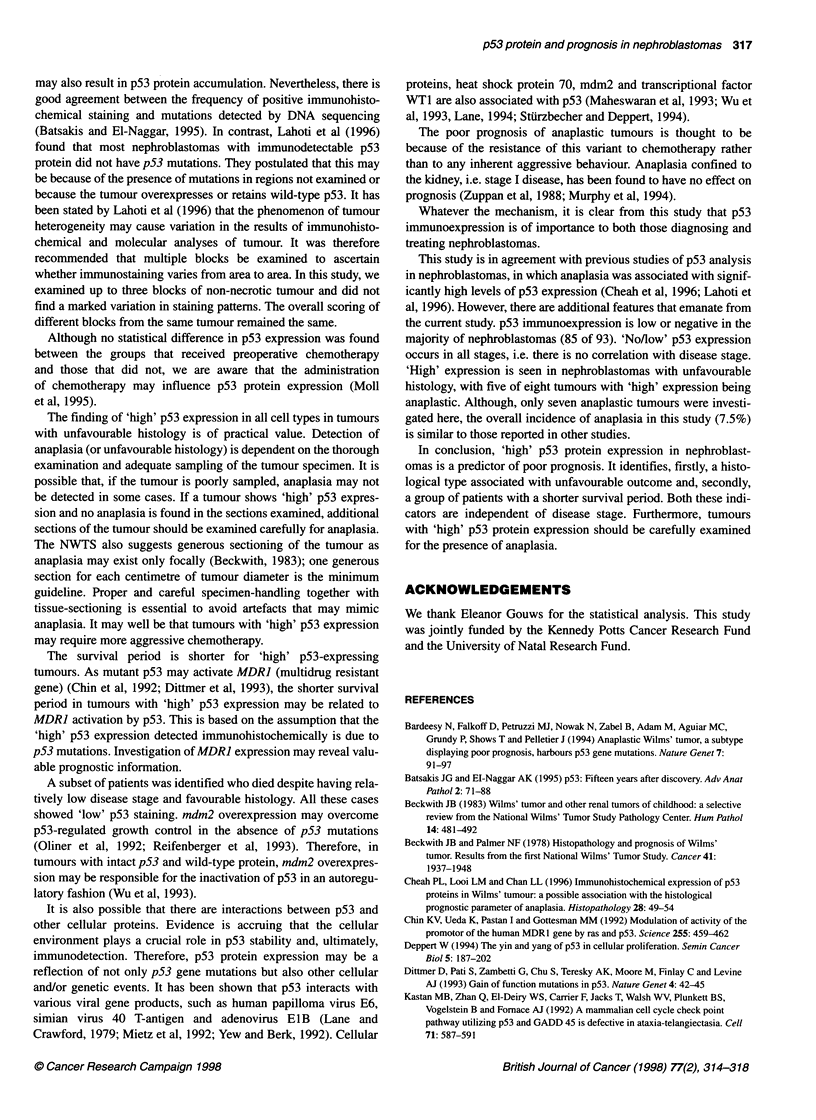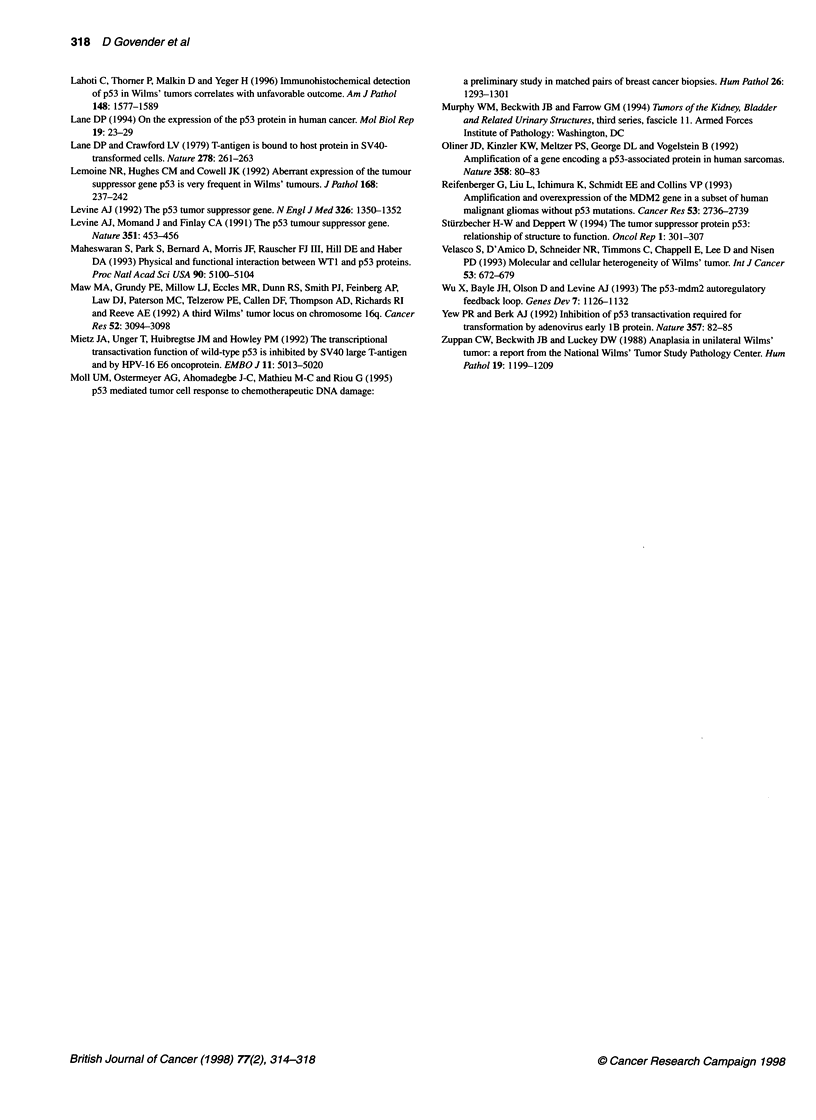# p53 protein expression in nephroblastomas: a predictor of poor prognosis.

**DOI:** 10.1038/bjc.1998.48

**Published:** 1998

**Authors:** D. Govender, P. Harilal, G. P. Hadley, R. Chetty

**Affiliations:** Department of Anatomical Pathology, University of Natal Medical School and King Edward VIII Hospital, Durban, South Africa.

## Abstract

**Images:**


British Journal of Cancer (1998) 77(2), 314–318
© 1998 Cancer Research Campaign

### p53 protein expression in nephroblastomas: a predictor of poor prognosis

**D Govender[1], P Harilal[1], GP Hadley[2] and R Chetty[1]**

Departments of [1]Anatomical Pathology and [2]Paediatric Surgery, University of Natal Medical School and King Edward VIII Hospital, Private Bag 7, Congella, 4013, Durban, South Africa

**Summary** Alteration of the tumour-suppressor gene *p53* is the commonest genetic change encountered in human malignant tumours. A study was undertaken to ascertain the prognostic value of p53 immunoexpression in nephroblastomas. A series of 93 consecutive cases was analysed. Archival formalin-fixed, paraffin wax-embedded tissue sections were stained with monoclonal anti-p53 antibody (DO-7, Dako) using a peroxidase-labelled streptavidin biotin kit. Five of seven tumours (71.4%) with unfavourable histology, but only 3 of 86 favourable histology tumours, showed 'high' p53 immunoexpression ($P < 0.001$). p53 expression in unfavourable histology tumours was present in both anaplastic and non-anaplastic components. Moreover, there was uniform staining of blastema, epithelium and stroma in unfavourable histology tumours. No statistical difference in p53 expression was found between patients who had received and those who had not received preoperative chemotherapy ($P = 0.678$). Similarly, no statistical difference was found in the groups of patients who were disease free, who had residual/recurrent disease or who had died ($P = 0.238$). The mean survival period for patients with tumours that had 'low' and 'high' expressions was 24.8 months and 12.6 months respectively ($P = 0.0003$). In conclusion, p53 immunoexpression in nephroblastomas was found to be an important determinant of poor prognosis as it identifies those patients with a shorter survival period and also those with unfavourable histology tumours. It may also be of practical value to the practising pathologist by identifying those tumours that require careful assessment for the presence of anaplasia.

**Keywords:** nephroblastoma; p53; Wilms' tumour

Nephroblastoma or Wilms' tumour is one of the commonest solid paediatric neoplasms with an incidence of approximately 1 in 10 000 children. There has been substantial improvement in the prognosis of patients with nephroblastoma largely as a result of the correct application of chemo- and radiotherapy.

The role of gene protein expression in determining prognosis is a relatively new investigative tool. The *p53* gene, located on the short arm of chromosome 17 (17p13), is the best known and most intensively studied tumour-suppressor gene in human cancer. Alterations in *p53* are among the most common genetic changes associated with human cancer, and this gene has been implicated in the oncogenesis of a wide range of neoplasms (Levine et al, 1991; Batsakis and El-Naggar, 1995).

Wild-type p53 maintains genomic integrity by functioning as a check point in the cell cycle at the $G_1$/S transition (Kastan et al, 1992; Levine, 1992). Alterations of the *p53* gene result in the formation of abnormal (mutant) p53 protein that has a longer half-life than the wild-type protein (Batsakis and El-Naggar, 1995). Mutant p53 protein may either lose its normal function or gain a new function that inactivates wild-type protein (Deppert, 1994).

Although immunohistochemical detection of p53 protein is neither a perfect nor a completely accurate reflection of underlying *p53* mutations, there is good agreement between positive immunohistochemical staining and mutations detected by DNA sequencing (Batsakis and El-Naggar, 1995).

*Received 25 February 1997*
*Revised 13 May 1997*
*Accepted 24 June 1997*

*Correspondence to:* D Govender

Alterations in the *p53* gene have been identified and implicated in the development of several human cancers, including colon, breast, lung and brain (Levine et al, 1991; Batsakis and El-Naggar, 1995). The *p53* gene in nephroblastomas was studied by Bardeesy and colleagues who found that *p53* mutations were restricted to anaplastic tumours only (Bardeesy et al, 1994). A few studies have looked at the immunohistochemical expression of p53 protein in nephroblastomas (Lemoine et al, 1992; Cheah et al, 1996) while Lahoti et al (1996) looked at both gene and protein, but these studies had small patient numbers and some had limited follow-up data.

This study was conducted on 93 cases of nephroblastomas to ascertain whether p53 protein immunoexpression is of prognostic value. p53 expression was correlated with the following parameters: histological classification, disease stage and survival period. In addition, this study represents the first analysis of this type on an African cohort of patients.

### MATERIALS AND METHODS

Ninety-three consecutive cases of nephroblastomas were retrieved from the files of the Department of Anatomical Pathology. The tumours were liberally sampled with an average of 20 blocks taken and were classified into favourable and unfavourable histology based on the presence or absence of anaplasia. Anaplasia was diagnosed when the following three criteria were present (Beckwith and Palmer, 1978): (1) an increase in nuclear diameter at least three times that of adjacent nuclei of the same cell type; (2) hyperchromatism of the enlarged nuclei; and (3) presence of abnormal mitotic figures.

**Figure 1** Histological appearance of a nephroblastoma with unfavourable histology: cells with bizarre, enlarged, hyperchromatic nuclei containing abnormal mitoses (haematoxylin and eosin, × 100)

**Figure 3** p53 immunostaining in an anaplastic area. Note intense nuclear labelling of all cells, including those that are large and pleomorphic (anti-p53, × 25)

**Figure 2** An example of 'low' p53 staining with fewer than 25% of cells stained (anti-p53, × 100)

**Table 1** p53 immunoexpression vs histological classification

| p53 immunoexpression | Histological classification | |
|---|---|---|
| | Favourable (%) | Unfavourable (%) |
| Low | 83 (96.5) | 2 (28.6) |
| High | 3 (3.5) | 5 (71.4) |

A minimum of two and maximum of three paraffin wax-embedded tissue blocks (those containing the least amount of tumour necrosis) were selected from each case for immunohistochemical staining. Tissue sections (2 µm) were placed on poly-L-lysine (Sigma diagnostics, St Louis, USA)-coated glass slides. Tissue sections immersed in a 0.01 M sodium citrate (trihydrate) solution were treated in a microwave (H2500 Microwave Processor, Energy Beam Sciences, Agawam, MA, USA) at 85°C for 10 min for antigen retrieval.

Endogenous peroxidase activity was blocked using an aqueous solution of 3% hydrogen peroxide. Sections were stained with monoclonal anti-p53 antibody (DO-7, Dako; dilution 1:100) using the peroxidase-labelled streptavidin biotin kit (Dako). The reaction was visualized using diaminobenzidine (Liquid DAB, Dako) as a chromogen. A case of poorly differentiated adenocarcinoma of the colon, known to be immunoreactive for p53 (DO-7), was used as a positive control. Negative controls, in which the primary antibody was withheld, were also run simultaneously.

Only crisp nuclear staining was accepted as positive. The quantity of nuclear staining was graded as follows: < 5%, negative; 6–25%, 1 +; 26–50%, 2 +; 51–75%, 3 +; and > 75%, 4 +. These cases were then arbitrarily divided into 'no/low' (0–50%) and 'high' (51–100%). The number of tumour cells that were immunopositive was expressed as a percentage of the total number of tumour cells per high-power field (HPF). One hundred HPFs were counted per slide and the final percentage was an average of the 100 HPFs. The quantitation and grading was performed manually using an Olympus BH2 microscope.

Clinical data were obtained from patient files kept in the Department of Paediatric Surgery. p53 protein expression was compared with clinicopathological stage, histological classification and patient outcome using Fisher's exact test and the chi-square test. The survival periods for grades of p53 expression were compared using Student's *t*-test.

### RESULTS

There were 49 female and 44 male patients with a F/M ratio of 1.1:1.0. The patient age at presentation ranged from 4 months to 14 years. The mean age at presentation was 3 years 7 months.

The first patient in this study was admitted on 13 January 1984 and the last patient on 4 May 1995, giving a time frame of approximately 11 years. The last patient in this study has been followed up for 12 months.

The overall follow-up period ranged from 1 month to 9 years 2 months, with a mean follow-up of 23.8 months. Fifty-six patients were disease free at last follow-up, 16 had residual or recurrent disease and 21 had died.

Sixteen patients had stage I disease, 24 had stage II, 23 had stage III, 27 had stage IV and three had stage V disease. There were 86 nephroblastomas with favourable histology and seven with unfavourable histology. Nephroblastomas with unfavourable histology (anaplastic) were identified by the presence of enlarged, hyperchromatic nuclei and multipolar mitotic figures (Figure 1). Sixty-eight patients received preoperative chemotherapy according to the SIOP (International Society of Paediatric Oncology) protocol. The remaining 25 patients did not receive preoperative chemotherapy.

© Cancer Research Campaign 1998

**Figure 4** Tubular structures in an anaplastic variety of nephroblastoma showing strong nuclear immunoreactivity for p53. Thus, in the anaplastic nephroblastomas, high p53 protein expression was also present in non-anaplastic ('favourable') areas (anti-p53, × 100)

**Table 2** p53 immunoexpression vs clinicopathological stage

| p53 immunoexpression | Clinicopathological stage | | | | |
|---|---|---|---|---|---|
| | I (%) | II (%) | III (%) | IV (%) | V (%) |
| Low | 16 (100) | 23 (95.8) | 20 (87.0) | 25 (92.6) | 1 (33.3) |
| High | 0 | 1 (4.2) | 3 (13) | 2 (7.4) | 2 (66.7) |

**Table 3** p53 immunoexpression vs disease status

| p53 immunoexpression | Disease status | | |
|---|---|---|---|
| | Disease free (%) | Alive with disease (%) | Died (%) |
| Low | 53 (94.6) | 13 (81.3) | 19 (90.5) |
| High | 3 (5.4) | 3 (18.7) | 2 (9.5) |

**Table 4** p53 immunoexpression vs survival

| p53 immunoexpression | Survival (months) | | | | |
|---|---|---|---|---|---|
| | < 6 (%) | 6–12 (%) | 12–18 (%) | 18–24 (%) | ≥ 24 (%) |
| Low | 13 (92.9) | 21 (91.3) | 14 (77.8) | 8 (88.9) | 29 (100) |
| High | 1 (7.1) | 2 (8.7) | 4 (22.2) | 1 (11.1) | 0 |

In total, 85 tumours showed 'no/low' p53 expression (Figure 2) and eight showed 'high' p53 expression. Normal kidney tissue, whenever present, did not show immunoreactivity for p53.

When comparing p53 expression with histology (see Table 1), five of the seven tumours (71.4%) with unfavourable histology showed 'high' p53 expression, while only 3 of 86 (3.5%) tumours with favourable histology showed similar expression. This difference was statistically significant ($P < 0.001$). Of the five tumours with unfavourable histology that showed 'high' p53 expression, four had diffuse anaplasia and the remaining one had focal anaplasia. 'High' p53 expression in tumours with unfavourable histology was present in both anaplastic (Figure 3) and non-anaplastic cells. Non-anaplastic tubular and/or glomeruloid structures, present in three of seven anaplastic tumours, showed intense nuclear staining (Figure 4). Furthermore, the 'high' expression in the anaplastic tumours was present uniformly in blastema, stroma and epithelium. Within the favourable histology group, three cases showed 'high' p53 expression. Two of these patients have recurrent disease and one patient died.

The results of p53 immunoexpression according to clinicopathological stage are shown in Table 2. All stage I tumours showed 'low' p53 staining; in fact, all 16 cases in this stage were either negative or showed less than 25% p53 immunostaining. There were 39 cases that had a combination of low disease stage (stage I and II) and favourable histology. All of these had 'low' p53 expression. Conversely, there were 46 (44 cases were either negative or showed 1 + p53 positivity) high-stage tumours also with 'low' p53 expression. This also included cases with favourable histology. This particular cohort of 46 cases had an overall survival of 23 months compared with 21.6 months survival of all high-stage nephroblastomas. Similarly, seven high-stage tumours with 'high' p53 levels were identified and this group had an overall survival of only 12.7 months. Although a statistically significant correlation was found between p53 immunoexpression and disease stage ($P = 0.004$), this result may not be valid because of the small number of patients with stage V tumours. Six patients with stage II disease, favourable histology and 'low' p53 staining died. Of these six patients, four died of treatment-related causes, e.g. infection due to bone marrow suppression, one died of recurrent tumour and another patient of unrelated causes.

Tumours from 7 of the 68 patients that received preoperative chemotherapy showed 'high' expression. In contrast, only one tumour of the 25 that were not treated preoperatively showed 'high' expression. No statistical difference in p53 staining was found between these two groups ($P = 0.678$).

Similarly, no statistical difference was found in p53 staining in the groups of patients who were disease free, who had residual/recurrent disease or who had died ($P = 0.238$) (Table 3). Tumours from 3 of the 56 patients who were disease free showed 'high' expression. In addition, 3 of the 16 who had residual/recurrent disease and 2 of the 21 who died showed 'high' expression.

The results of p53 immunoexpression versus survival are shown in Table 4. The mean survival period for patients with tumours that showed 'low' expression was 24.8 months (range 1–110 months). In contrast, the mean survival period for the 'high' expression group was 12.6 months (range 2–19 months). This finding was statistically significant ($P = 0.0003$, Student's $t$-test).

### DISCUSSION

Several genetic aberrations have been implicated in the pathogenesis of nephroblastomas. These include the following loci: 11p13, 11p15 and 16q (Maw et al, 1992). The most widely investigated is the *WT1* gene, which is mapped to 11p13. The *p53* gene has also been suggested to play a role in the pathogenesis of nephroblastomas (Lemoine et al, 1992; Velasco et al, 1993).

This study looked at the prognostic significance of the immunohistochemical detection of p53 protein in nephroblastomas. It should be emphasized that the immunohistochemical detection of p53 protein is neither a perfect nor an accurate reflection of underlying *p53* mutations, because mechanisms other than mutations

 © Cancer Research Campaign 1998

may also result in p53 protein accumulation. Nevertheless, there is good agreement between the frequency of positive immunohistochemical staining and mutations detected by DNA sequencing (Batsakis and El-Naggar, 1995). In contrast, Lahoti et al (1996) found that most nephroblastomas with immunodetectable p53 protein did not have *p53* mutations. They postulated that this may be because of the presence of mutations in regions not examined or because the tumour overexpresses or retains wild-type p53. It has been stated by Lahoti et al (1996) that the phenomenon of tumour heterogeneity may cause variation in the results of immunohistochemical and molecular analyses of tumour. It was therefore recommended that multiple blocks be examined to ascertain whether immunostaining varies from area to area. In this study, we examined up to three blocks of non-necrotic tumour and did not find a marked variation in staining patterns. The overall scoring of different blocks from the same tumour remained the same.

Although no statistical difference in p53 expression was found between the groups that received preoperative chemotherapy and those that did not, we are aware that the administration of chemotherapy may influence p53 protein expression (Moll et al, 1995).

The finding of 'high' p53 expression in all cell types in tumours with unfavourable histology is of practical value. Detection of anaplasia (or unfavourable histology) is dependent on the thorough examination and adequate sampling of the tumour specimen. It is possible that, if the tumour is poorly sampled, anaplasia may not be detected in some cases. If a tumour shows 'high' p53 expression and no anaplasia is found in the sections examined, additional sections of the tumour should be examined carefully for anaplasia. The NWTS also suggests generous sectioning of the tumour as anaplasia may exist only focally (Beckwith, 1983); one generous section for each centimetre of tumour diameter is the minimum guideline. Proper and careful specimen-handling together with tissue-sectioning is essential to avoid artefacts that may mimic anaplasia. It may well be that tumours with 'high' p53 expression may require more aggressive chemotherapy.

The survival period is shorter for 'high' p53-expressing tumours. As mutant p53 may activate *MDR1* (multidrug resistant gene) (Chin et al, 1992; Dittmer et al, 1993), the shorter survival period in tumours with 'high' p53 expression may be related to *MDR1* activation by p53. This is based on the assumption that the 'high' p53 expression detected immunohistochemically is due to *p53* mutations. Investigation of *MDR1* expression may reveal valuable prognostic information.

A subset of patients was identified who died despite having relatively low disease stage and favourable histology. All these cases showed 'low' p53 staining. *mdm2* overexpression may overcome p53-regulated growth control in the absence of *p53* mutations (Oliner et al, 1992; Reifenberger et al, 1993). Therefore, in tumours with intact *p53* and wild-type protein, *mdm2* overexpression may be responsible for the inactivation of p53 in an autoregulatory fashion (Wu et al, 1993).

It is also possible that there are interactions between p53 and other cellular proteins. Evidence is accruing that the cellular environment plays a crucial role in p53 stability and, ultimately, immunodetection. Therefore, p53 protein expression may be a reflection of not only *p53* gene mutations but also other cellular and/or genetic events. It has been shown that p53 interacts with various viral gene products, such as human papilloma virus E6, simian virus 40 T-antigen and adenovirus E1B (Lane and Crawford, 1979; Mietz et al, 1992; Yew and Berk, 1992). Cellular proteins, heat shock protein 70, mdm2 and transcriptional factor WT1 are also associated with p53 (Maheswaran et al, 1993; Wu et al, 1993, Lane, 1994; Stürzbecher and Deppert, 1994).

The poor prognosis of anaplastic tumours is thought to be because of the resistance of this variant to chemotherapy rather than to any inherent aggressive behaviour. Anaplasia confined to the kidney, i.e. stage I disease, has been found to have no effect on prognosis (Zuppan et al, 1988; Murphy et al, 1994).

Whatever the mechanism, it is clear from this study that p53 immunoexpression is of importance to both those diagnosing and treating nephroblastomas.

This study is in agreement with previous studies of p53 analysis in nephroblastomas, in which anaplasia was associated with significantly high levels of p53 expression (Cheah et al, 1996; Lahoti et al, 1996). However, there are additional features that emanate from the current study. p53 immunoexpression is low or negative in the majority of nephroblastomas (85 of 93). 'No/low' p53 expression occurs in all stages, i.e. there is no correlation with disease stage. 'High' expression is seen in nephroblastomas with unfavourable histology, with five of eight tumours with 'high' expression being anaplastic. Although, only seven anaplastic tumours were investigated here, the overall incidence of anaplasia in this study (7.5%) is similar to those reported in other studies.

In conclusion, 'high' p53 protein expression in nephroblastomas is a predictor of poor prognosis. It identifies, firstly, a histological type associated with unfavourable outcome and, secondly, a group of patients with a shorter survival period. Both these indicators are independent of disease stage. Furthermore, tumours with 'high' p53 protein expression should be carefully examined for the presence of anaplasia.

### ACKNOWLEDGEMENTS

We thank Eleanor Gouws for the statistical analysis. This study was jointly funded by the Kennedy Potts Cancer Research Fund and the University of Natal Research Fund.

### REFERENCES

Bardeesy N, Falkoff D, Petruzzi MJ, Nowak N, Zabel B, Adam M, Aguiar MC, Grundy P, Shows T and Pelletier J (1994) Anaplastic Wilms' tumor, a subtype displaying poor prognosis, harbours p53 gene mutations. *Nature Genet* **7**: 91–97

Batsakis JG and El-Naggar AK (1995) p53: Fifteen years after discovery. *Adv Anat Pathol* **2**: 71–88

Beckwith JB (1983) Wilms' tumor and other renal tumors of childhood: a selective review from the National Wilms' Tumor Study Pathology Center. *Hum Pathol* **14**: 481–492

Beckwith JB and Palmer NF (1978) Histopathology and prognosis of Wilms' tumor. Results from the first National Wilms' Tumor Study. *Cancer* **41**: 1937–1948

Cheah PL, Looi LM and Chan LL (1996) Immunohistochemical expression of p53 proteins in Wilms' tumour: a possible association with the histological prognostic parameter of anaplasia. *Histopathology* **28**: 49–54

Chin KV, Ueda K, Pastan I and Gottesman MM (1992) Modulation of activity of the promotor of the human MDR1 gene by ras and p53. *Science* **255**: 459–462

Deppert W (1994) The yin and yang of p53 in cellular proliferation. *Semin Cancer Biol* **5**: 187–202

Dittmer D, Pati S, Zambetti G, Chu S, Teresky AK, Moore M, Finlay C and Levine AJ (1993) Gain of function mutations in p53. *Nature Genet* **4**: 42–45

Kastan MB, Zhan Q, El-Deiry WS, Carrier F, Jacks T, Walsh WV, Plunkett BS, Vogelstein B and Fornace AJ (1992) A mammalian cell cycle check point pathway utilizing p53 and GADD 45 is defective in ataxia-telangiectasia. *Cell* **71**: 587–591

© Cancer Research Campaign 1998

Lahoti C, Thorner P, Malkin D and Yeger H (1996) Immunohistochemical detection of p53 in Wilms' tumors correlates with unfavorable outcome. *Am J Pathol* **148**: 1577–1589

Lane DP (1994) On the expression of the p53 protein in human cancer. *Mol Biol Rep* **19**: 23–29

Lane DP and Crawford LV (1979) T-antigen is bound to host protein in SV40-transformed cells. *Nature* **278**: 261–263

Lemoine NR, Hughes CM and Cowell JK (1992) Aberrant expression of the tumour suppressor gene p53 is very frequent in Wilms' tumours. *J Pathol* **168**: 237–242

Levine AJ (1992) The p53 tumor suppressor gene. *N Engl J Med* **326**: 1350–1352

Levine AJ, Momand J and Finlay CA (1991) The p53 tumour suppressor gene. *Nature* **351**: 453–456

Maheswaran S, Park S, Bernard A, Morris JF, Rauscher FJ III, Hill DE and Haber DA (1993) Physical and functional interaction between WT1 and p53 proteins. *Proc Natl Acad Sci USA* **90**: 5100–5104

Maw MA, Grundy PE, Millow LJ, Eccles MR, Dunn RS, Smith PJ, Feinberg AP, Law DJ, Paterson MC, Telzerow PE, Callen DF, Thompson AD, Richards RI and Reeve AE (1992) A third Wilms' tumor locus on chromosome 16q. *Cancer Res* **52**: 3094–3098

Mietz JA, Unger T, Huibregtse JM and Howley PM (1992) The transcriptional transactivation function of wild-type p53 is inhibited by SV40 large T-antigen and by HPV-16 E6 oncoprotein. *EMBO J* **11**: 5013–5020

Moll UM, Ostermeyer AG, Ahomadegbe J-C, Mathieu M-C and Riou G (1995) p53 mediated tumor cell response to chemotherapeutic DNA damage: a preliminary study in matched pairs of breast cancer biopsies. *Hum Pathol* **26**: 1293–1301

Murphy WM, Beckwith JB and Farrow GM (1994) *Tumors of the Kidney, Bladder and Related Urinary Structures*, third series, fascicle 11. Armed Forces Institute of Pathology: Washington, DC

Oliner JD, Kinzler KW, Meltzer PS, George DL and Vogelstein B (1992) Amplification of a gene encoding a p53-associated protein in human sarcomas. *Nature* **358**: 80–83

Reifenberger G, Liu L, Ichimura K, Schmidt EE and Collins VP (1993) Amplification and overexpression of the MDM2 gene in a subset of human malignant gliomas without p53 mutations. *Cancer Res* **53**: 2736–2739

Stürzbecher H-W and Deppert W (1994) The tumor suppressor protein p53: relationship of structure to function. *Oncol Rep* **1**: 301–307

Velasco S, D'Amico D, Schneider NR, Timmons C, Chappell E, Lee D and Nisen PD (1993) Molecular and cellular heterogeneity of Wilms' tumor. *Int J Cancer* **53**: 672–679

Wu X, Bayle JH, Olson D and Levine AJ (1993) The p53-mdm2 autoregulatory feedback loop. *Genes Dev* **7**: 1126–1132

Yew PR and Berk AJ (1992) Inhibition of p53 transactivation required for transformation by adenovirus early 1B protein. *Nature* **357**: 82–85

Zuppan CW, Beckwith JB and Luckey DW (1988) Anaplasia in unilateral Wilms' tumor: a report from the National Wilms' Tumor Study Pathology Center. *Hum Pathol* **19**: 1199–1209

*© Cancer Research Campaign 1998*